# Supplementary material for: Daily Life Patterns, Psychophysical Conditions, and Immunity of Adolescents in the COVID-19 Era: A Mixed Research with Qualitative Interviews by a Quasi-Experimental Retrospective Study
Source: Healthcare (Basel). 2022 Jun 20;10(6):1152. doi: 10.3390/healthcare10061152 (PMC9222600; doi:10.3390/healthcare10061152)
Supplement: Supplementary file 1 [file healthcare-10-01152-s001.zip › healthcare-1707987-supplementary.pdf]

Supplementary Materials

Table S1. Questionnaire components of daily life pattern.

	Questions	Answers	Related Variables
Q1	How healthy do you think you are?	1. I consider myself very unhealthy.<br>2. I consider myself unhealthy.<br>3. I think my health is average.<br>4. I consider myself healthy.<br>5. I consider myself very healthy.	Health
Q2	Are you prone to respiratory diseases such as colds?	1. I always get colds.<br>2. I am prone to colds.<br>3. I have average proneness to colds.<br>4. I am not prone to colds.<br>5. I never get colds.	
Q3	How many hours per day do you sleep on average?	1. Less than 5 hours<br>2. Between 5~6 hours<br>3. Between 6~7 hours<br>4. Between 7~8 hours<br>5. More than 8 hours	Sleep
Q4	What time do you usually sleep?	1. Before 21 o'clock<br>2. 21~22 o'clock<br>3. 22~23 o'clock<br>4. 23~24 o'clock<br>5. After 24 o'clock	
Q5	What time do you usually wake up?	1. Before 4 o'clock<br>2. 4~5 o'clock<br>3. 5~6 o'clock<br>4. 6~7 o'clock<br>5. 7~8 o'clock<br>6. After 8 o'clock	
Q6	How many meals do you eat per day?	1. One time<br>2. Two times<br>3. Three times<br>4. Four or more times	Diet
Q7	How many days a week do you exercise for at least 30 minutes a day?	1. None<br>2. One day<br>3. Two days<br>4. Three days<br>5. More than four days	Exercise

Table S2. Comparative results of CES-D among the four groups.

.	Groups						
	**NDG**	**LDG**	**MDG**	**HDG**	**F**	***p***	***η*²**
CES-D 1	0.51 ± 0.73	1.04 ± 1.15	1.33 ± 0.90	2.50 ± 0.76	15.075	0.001	0.288
CES-D 2	0.30 ± 0.72	0.43 ± 0.73	0.33 ± 0.82	0.75 ± 1.04	0.850	0.469	0.022
CES-D 3	0.08 ± 0.33	0.48 ± 0.67	0.73 ± 0.88	1.88 ± 1.36	22.846	0.001	0.380
CES-D 4	2.37 ± 1.03	2.13 ± 1.06	2.20 ± 0.86	1.00 ± 1.07	3.979	0.010	0.096
CES-D 5	0.52 ± 0.83	0.83 ± 0.78	0.87 ± 0.92	1.88 ± 0.99	6.450	0.001	0.147
CES-D 6	0.00 ± 0.00	1.00 ± 0.00	2.00 ± 0.00	3.00 ± 0.00	7.898	0.000	1.000
CES-D 7	0.87 ± 0.92	0.87 ± 0.81	0.93 ± 0.88	1.50 ± 1.20	1.400	0.247	0.036
CES-D 8	1.97 ± 0.99	2.09 ± 1.00	2.07 ± 0.88	2.13 ± 0.99	0.287	0.834	0.008
CES-D 9	0.27 ± 0.63	0.43 ± 0.59	0.27 ± 0.46	1.75 ± 1.28	11.407	0.001	0.234
CES-D 10	0.15 ± 0.47	0.65 ± 0.71	1.13 ± 1.13	2.00 ± 1.20	22.237	0.001	0.373
CES-D 11	0.32 ± 0.60	0.48 ± 0.73	0.80 ± 1.15	1.13 ± 1.36	3.519	0.017	0.086
CES-D 12	0.94 ± 0.97	1.04 ± 0.77	1.20 ± 0.77	1.88 ± 0.99	2.845	0.041	0.071
CES-D 13	0.20 ± 0.52	0.35 ± 0.65	0.87 ± 0.99	1.63 ± 1.30	12.174	0.001	0.246
CES-D 14	0.21 ± 0.50	0.74 ± 1.05	1.20 ± 1.01	1.63 ± 1.41	12.944	0.001	0.257
CES-D 15	0.13 ± 0.38	0.30 ± 0.47	0.40 ± 0.74	1.00 ± 1.31	6.036	0.001	0.139
CES-D 16	1.27 ± 1.07	1.30 ± 0.70	1.67 ± 0.98	1.00 ± 1.20	0.920	0.434	0.024
CES-D 17	0.14 ± 0.49	0.48 ± 0.79	0.27 ± 0.59	1.50 ± 1.20	10.314	0.001	0.216
CES-D 18	0.10 ± 0.34	0.52 ± 0.90	0.60 ± 0.63	1.63 ± 1.30	15.652	0.001	0.295
CES-D 19	0.14 ± 0.35	0.30 ± 0.56	0.33 ± 0.62	1.13 ± 1.36	6.794	0.001	0.154
CES-D 20	0.32 ± 0.73	0.70 ± 0.82	0.73 ± 0.96	1.63 ± 1.06	6.773	0.001	0.154

All data represents mean ± standard deviation. Symbols [a], [b], [c], and [d] represent post hoc results from Bonferroni test. NDG, no-depression group; LDG, low-depression group; MDG, moderate-depression group; HDG, high-depression group.
